# The effects of superheated steam roasting on proximate analysis, antioxidant activity, and oil quality of black seed (*Nigella sativa*)

**DOI:** 10.1002/fsn3.3655

**Published:** 2023-09-06

**Authors:** K. V. Harivaindaran, Nguyễn Hữu Tiến, Toàn Nguyễn Song Đinh, Hayati Samsudin, Fazilah Ariffin, Abdorreza Mohammadi Nafchi

**Affiliations:** ^1^ Food Technology Division, School of Industrial Technology Universiti Sains Malaysia Penang Malaysia; ^2^ Food Biopolymer Research Group, Food Science and Technology Department, Damghan Branch Islamic Azad University Damghan Iran; ^3^ Green Biopolymer, Coatings & Packaging Cluster, School of Industrial Technology Universiti Sains Malaysia Penang Malaysia

**Keywords:** antioxidant, black cumin, *Nigella sativa*, oil analysis, superheated steam

## Abstract

*Nigella sativa*, commonly known as the black seed, is a culinary spice therapeutic against many ailments. Common preparation practice of roasting or heating the seeds often deteriorates bioactive compounds, which can be remedied with superheated steam (SHS). With roasting temperatures of 150, 200, and 250°C and roasting times of 10, 15, and 20 min, convection and SHS roasting media were tested, and their effects on proximate analysis, antioxidant assays, and oil quality were evaluated. For proximate content, moisture significantly decreased from 9.08% in unroasted seeds to 4.18%–1.04% in roasted seeds, while fat increased to as high as 44.76% from 32.87% in unroasted seeds. Roasting only slightly increased ash content and had no significant impact on protein and carbohydrate content. SHS roasted black seeds had better DPPH (2,2‐Diphenyl‐1‐picrylhydrazyl) radical scavenging capacity (RSC) than convection roasted seeds. DPPH RSC decreased with elevated roasting time and temperature, conversely related to total phenolic content, which increased with increased roasting time and temperature. Oil of roasted seeds developed an increasingly intense brown color from an initial light, yellow, unroasted oil with better extraction efficiency in SHS roasting. For oil quality analysis, free fatty acid values were significantly lower in both roasted samples. Peroxide value was initially recorded at 84 in convection and 48 (meq O_2_/kg of oil) in SHS roasted samples. In contrast, p‐anisidine values were initially recorded at 28.36 in convection roasted samples compared to 23.73 in SHS roasted samples. Based on all quality analyses, SHS showed better potential in black seed quality preservation.

## Graphical abstract

With roasting temperatures of 150, 200, and 250°C and roasting times of 10, 15, and 20 min, convection and superheated steam (SHS) roasting media were tested, and their effects on proximate analysis, antioxidant assays, and oil quality were evaluated. SHS roasted black seeds had better 2,2‐diphenyl‐1‐picrylhydrazyl (DPPH) radical scavenging capacity (RSC) than convection roasted seeds. Based on all quality analyses, SHS showed better black seed quality preservation potential.

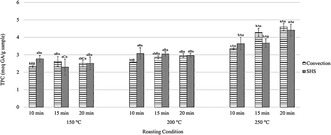

## 1 INTRODUCTION

**FIGURE 1:**
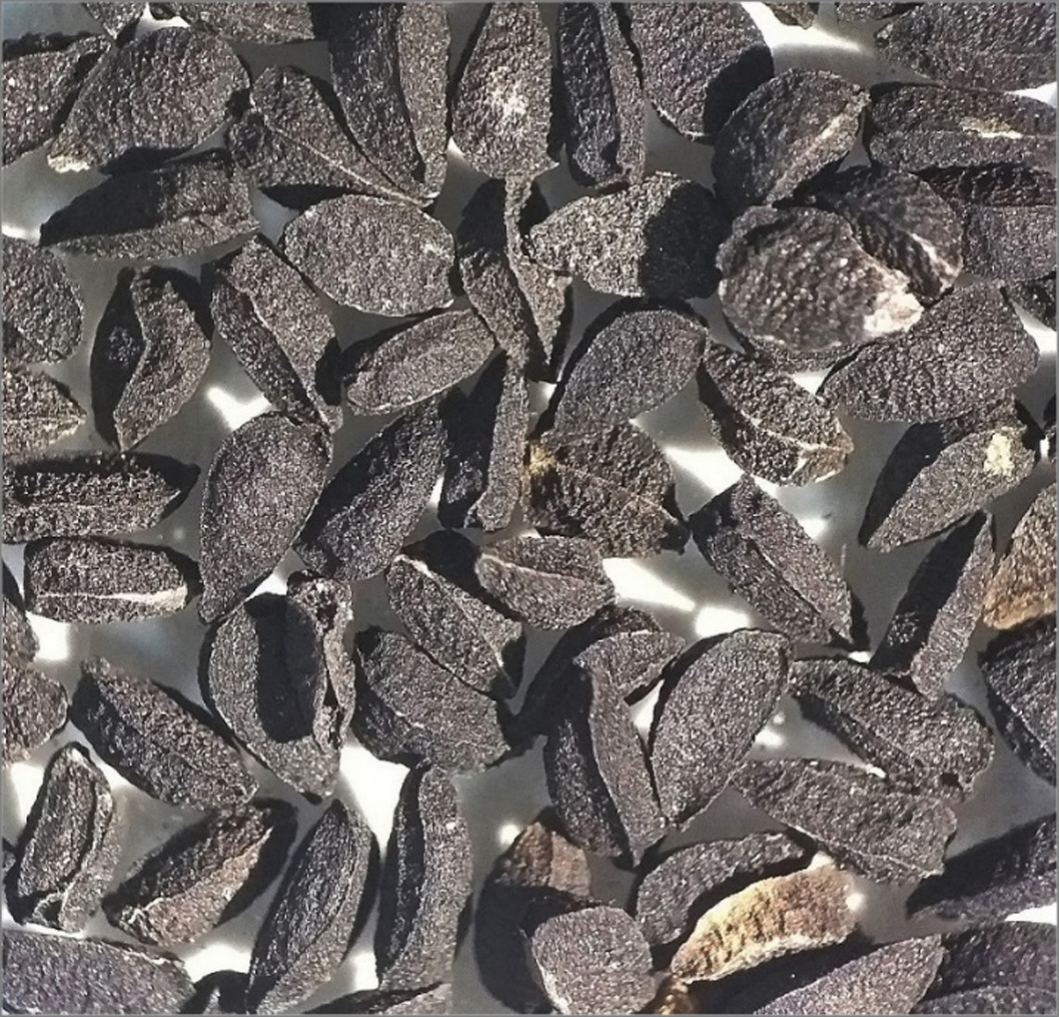
*Nigella sativa* seeds observed under a stereomicroscope.

Belonging to the family Ranunculaceae, *Nigella sativa* L. seeds possess a world of therapeutic potency, including its ability to ease asthma, hypertension, diabetes, inflammation, cough, bronchitis, headache, eczema, fever, dizziness, and influenza (Ghiasi et al., 2022; Ramadan, 2007). A consumer favorite, black seed oil is an economically viable constituent of the seed and is gaining popularity worldwide (Anshiso & Teshome, 2018; Hadi et al., 2021; Figure 1).

Roasting black seeds is a relatively unexplored avenue in the food industry and in the scientific world, whereby research on different roasting techniques and parameters has been surfacing only within the past decade (Agbaria et al., 2015; Jan et al., 2019; Liang et al., 2018; Mazaheri et al., 2019; Suri et al., 2022). Besides packing ample phytonutrients, black seeds are a potential candidate to be roasted since many cultural practices around the world involve heating them in some way, for example, being baked into Turkish bread (Kiralan, 2012) and in roasted Indian spice mix, and pickling and braising meat (Malhotra, 2012). Roasting oilseeds before acquiring their lipid fraction adds a characteristic flavor that can be organoleptically pleasing, almost necessitating it (Lee et al., 2010). Organoleptic goals aside, roasting also allows the expression of compounds that dispense health benefits upon intake while aiding the elimination or reduction in microbes and toxins (Sruthi et al., 2021).

Unfortunately, health benefits of black seed can be thwarted by heating, resulting in the loss of important bioactive volatile compounds (Kiralan, 2012). This phenomenon is a result of thermal degradation that negatively affects heat susceptible compounds by effectively reducing value of roasted products (Shan et al., 2015). The loss of these bioactive compounds also allows for lipid oxidation to occur more freely (Perren & Escher, 2013). Moreover, heat from roasting also induces lipid oxidation causing the formation of hydroperoxides which are compounds that can cause rancidity in oils (Yaacoub et al., 2008). Since more traditional roasting methods can be damaging to the resulting oil, means of achieving similar outcomes in flavor, quality, and yield with better roasting media are important, one of them being superheated steam (SHS; Darvish et al., 2022; Liang et al., 2018; Sruthi et al., 2021). SHS is dry steam produced by applying sensible heat to wet steam, raising steam temperature to higher than 100°C (Head et al., 2010). SHS operates in very low‐to‐nil oxygen saturation, whereby oxidation or reactions susceptible to ignition are evaded or reduced (Mujumdar, 2014). The absence of oxygen during heat treatment tends to result in better‐quality end products containing important bioactive compounds that add value to the end product (Mujumdar & Law, 2010).

SHS has been proven to preserve the antioxidant prowess of cocoa when roasted within a temperature range of 150–250°C, and a roasting time range between 10 and 50 min showed significant preservation of cocoa antioxidant capacity when compared to convection roasting (Zzaman et al., 2013). Similarly, coffee roasted with SHS at 200°C for 20 min had significantly superior antioxidant properties than unroasted coffee (Shan et al., 2015). Even black seeds roasted with SHS at 180°C for 10, 15, and 20 min showed significantly increased antioxidant content (Liang et al., 2018). Previous reports of SHS roasted black seeds lack information beyond proximate content and antioxidant analysis. Information on the oil quality of roasted black seeds is just as scarce. Hence, this paper details, in addition to the effects of roasting on black seeds, differences between SHS and convection roasting at varying roasting temperatures (150–250°C) and at different roasting times (10–20 min), with regards to proximate content, antioxidant capacity, quality of the resulting black seed oil, and effects on its essential oil.

## 2 MATERIALS AND METHODS

### 2.1 Materials

Analytical‐grade chemicals, 2‐propanol, acetic acid glacial, isooctane, petroleum ether, potassium iodide, sodium carbonate, sodium hydroxide, and sulfuric acid, were purchased from Orec. Kjeldahl tablet, p‐anisidine, and sodium thiosulfate were purchased from Merck. HPLC‐grade methanol was purchased from HmbG Chemicals. Boric acid and hydrochloric acid were purchased from Ever Gainful Enterprise. 2,2‐Diphenyl‐1‐picrylhydrazyl (DPPH), Folin Ciocalteu reagent, and gallic acid were purchased from Sigma Aldrich. Chloroform and phenolphthalein were purchased from R&M Chemicals.

### 2.2 Roasting

Black seeds procured from local vendors were air‐tight sealed and stored at room temperature until roasting was carried out. Vendors verified that the seeds were of Indian origin. Seventy grams of seeds were roasted with convection and SHS using a SHARP Healsio AX‐1500V oven (Japan) at 150, 200, and 250°C for 10, 15, and 20 min at each temperature. Temperature and time parameters were modified from Liang et al.'s work (2018), and Idrus et al.'s work (2017). Roasted black seeds were allowed to cool down completely before being ground for approximately 1 min using a coffee grinder and stored at room temperature in air‐tight conditions. Ground black seeds were later analyzed for proximate, antioxidant, and oil quality analyses. Roasting for proximate, antioxidant, and oil quality analyses was done in triplicates. Separately, 90 g of seeds were roasted and ground for essential oil extraction and qualitative analysis.

### 2.3 Proximate analysis

Proximate analysis was carried out according to AOAC's official Method of Analysis (AOAC, 2005). Moisture content was estimated gravimetrically via drying (AOAC, 935.29). Ten grams of roasted, ground black seeds were dried in a 105°C oven overnight, then weighed and returned to oven in hourly intervals until a constant weight was achieved. Moisture was calculated based on difference in weight. Fat was determined via Soxhlet extraction using petroleum ether as solvent (AOAC, 963.15). Five grams of roasted and dried ground seeds were weighed into a thimble and placed into a Soxhlet extractor fitted with a preweighed flask and refluxed for approximately 4 h at approximately 50 ± 5°C. Extracted fat was dried in an oven and weighed in intervals until a constant was achieved to determine fat content. Protein was determined using the Kjeldahl method, and digestion was done using sulfuric acid (AOAC, 950.48). 0.3 g dried, defatted black seed powder was weighed with one 10 mg Kjeldahl tablet and 10 mL concentrated sulfuric acid and digested for an hour. Ten milliliter distilled water was added after the tube was allowed to cool down, then 35% NaOH was added in a protein distillation unit. Distillate was collected with boric acid and titrated with 0.02 M hydrochloric acid using methyl red indicator. Protein content was determined by volume difference of hydrochloric acid. Ash was determined by combustion in a furnace and determined gravimetrically (AOAC, 923.03). Three grams of sample were burned in a weighed crucible until black before being placed in a furnace at 550°C until no black particles were visible. Ash content was determined by the difference in weight. Carbohydrate content was calculated by difference as follows:

$$\%\text{Carbohydrate}=100-\left(\%\text{Moisture}+\%\mathrm{Fat}+\%\text{Crude protein}+\%\mathrm{Ash}\right)$$

### 2.4 Antioxidant analysis of roasted black seeds

Extraction for antioxidant assays was done according to Brodowska et al. (2014), with some modifications. Modifications to extraction are as follows; briefly, 10 mL methanol:water mixture (60:40, v/v) was added to 1 g of ground roasted black seed and rotated in a rotary benchtop shaker at 100 rotations per minute. The extract was defatted with 5 mL n‐hexane and centrifuged at 3500 rpm for 30 min. The hexane layer was discarded, and the resulting extract was used for antioxidant analysis. Antioxidant assays 2,2‐diphenyl‐1‐picrylhydrazyl (DPPH) radical scavenging capacity (RSC), expressed as % inhibition, and total phenolic content (TPC), expressed as mg gallic acid equivalent per g (mg/g), were carried out according to Kalantzakis et al. (2006).

### 2.5 Oil extraction and oil quality analysis

Oil extraction was done via solvent extraction with 30 g of ground black seeds, according to Bligh & Dyer (1959), with some modifications. Oil yield was determined in weight percentage (w/w%) according to Abubakar et al. (2012). Oil color was analyzed via colorimeter Minolta CM‐3500D (USA) equipped with SpectraMagic and expressed as L*, a*, and b*, based on Zzaman et al. (2014). Free fatty acids (FFA) analysis and peroxide value were estimated according to Official Methods of Analysis (AOAC, 2005). p‐ANISIDINE value was measured according to the American Oil Chemists Society (AOCS, 1998).

### 2.6 Qualitative analysis of essential oils (steam distillation and gas chromatography)

Roasted black seeds were ground and extracted for essential oil via hydrodistillation, according to Zelelew and Gebremariam (2018), with adjustments using a modified Clevenger's apparatus. A total volume of 400 mL distilled water was added to roasted and ground black seeds and heated at approximately 90°C (±5°C) for about 90 min. Distillate containing the essential oil layer was obtained using chloroform and a separating funnel. After evaporation of the organic solvent, the resulting essential oil was profiled via gas chromatography according to Gerige et al. (2009) with some modifications. The essential oil was dissolved in methanol and filtered using a 0.45 μm nylon syringe filter. One microliter of the mixture was injected into a GC 2010 Plus (Shimadzu) gas chromatograph equipped with an AOC 5000 Plus (Shimadzu) autosampler under split mode (1:33). 30 m BPX5 capillary column (i.d, 0.25 μm) with 0.25 μm film thickness was used. Chromatograph was programmed at an initial temperature of 70°C, held for 5 min, and set to an end temperature of 230°C, increased at a rate of 2°C/min. The end temperature was held for 10 min. The injector temperature was set at 230°C. Helium as a carrier gas was set at a 1 mL/min flow rate. Compounds were identified via Mass Spectra based on FFNSCI.3.LIB, WILEY229.LIB, and NIST08.LIB. Results were presented as percentage area (%).

### 2.7 Statistical analysis

Statistical analysis was done using SPSS version 26 via two‐way analysis of variance (ANOVA) at a significance level of *p* < .05. Multiple comparisons were made to establish a difference in means via the Tukey test.

## 3 RESULTS AND DISCUSSION

Moisture, fat, protein, ash, and carbohydrate contents are necessary for the basic building blocks of food products and for reflecting changes that occur within different processes involved (Qayyum et al., 2012).

Unroasted black seeds had a moisture content of 9.08 ± 0.15%. Several sources reported different values for untreated black seed moisture content (3.82%–19.04%) (Kumbhakar et al., 2017; Mukherjee & Datta, 2011; Oubannin et al., 2022; Suri et al., 2019). These variations depend on cultivation region and phenotype, among many other factors (Mamun & Absar, 2018). Roasting as a cooking method is known to reduce moisture content of black seeds (Mazaheri et al., 2019), as evidenced from the results in Table 1. It was further demonstrated by the fact that all samples had significantly different moisture content than an unroasted sample. Roasting at just 150°C for 10 min (at the lowest temperature and shortest time) drastically reduced moisture content (approximately 66% moisture loss). Although SHS is a supposedly efficient dry gas (Head et al., 2010), results indicated no discernible significance between both roasting media in terms of moisture loss. Nonetheless, both roasting media caused a significant reduction in moisture which lends to extending the shelf life of food products (Varastegani et al., 2018).

**TABLE 1:** Proximate content (%) of black seeds roasted via convection and SHS.

Proximate content	Roasting medium	Temp (°C)	Time (min)		
			10	15	20
Moisture	Convection	150	3.04 ± 0.17^aAα^	2.27 ± 0.09^bAα^	2.02 ± 0.06^cAα^
		200	2.29 ± 0.10^aBβ^	1.67 ± 0.04^bBβ^	1.35 ± 0.09^cBβ^
		250	1.57 ± 0.16^aCα^	1.24 ± 0.02^bCβ^	1.15 ± 0.08^bCα^
	SHS	150	3.06 ± 0.16^aAα^	2.28 ± 0.08^bAα^	2.04 ± 0.09^cAα^
		200	2.71 ± 0.08^aBα^	2.08 ± 0.19^bAα^	1.71 ± 0.22^cAα^
		250	1.28 ± 0.04^aCβ^	1.25 ± 0.02^aBα^	1.08 ± 0.09^aBβ^
Fat	Convection	150	34.39 ± 1.69^aBα^	35.80 ± 1.02^aBα^	36.59 ± 2.37^aBα^
		200	34.68 ± 1.38^bBα^	36.45 ± 1.30^abBα^	37.96 ± 1.27^aBα^
		250	39.92 ± 1.10^bAα^	41.53 ± 2.37^bAα^	44.76 ± 1.10^aAα^
	SHS	150	33.92 ± 1.02^bBα^	33.58 ± 1.33^bCα^	36.40 ± 1.03^aBα^
		200	34.08 ± 0.99^bBα^	35.78 ± 1.11^abBα^	37.02 ± 1.40^aBα^
		250	37.81 ± 1.29^bAα^	40.67 ± 0.71^aAα^	42.36 ± 0.65^aAβ^
Protein	Convection	150	21.58 ± 0.59^aAα^	21.51 ± 0.96^aAα^	19.88 ± 2.20^aAα^
		200	21.40 ± 1.23^aAα^	20.57 ± 1.49^aAα^	20.06 ± 0.89^aAα^
		250	22.69 ± 1.71^aAα^	20.66 ± 0.60^abAβ^	19.19 ± 1.16^bAβ^
	SHS	150	21.41 ± 1.10^aBα^	19.80 ± 0.40^abBα^	19.54 ± 0.81^bBα^
		200	21.33 ± 0.54^aBα^	20.31 ± 1.16^aBα^	20.02 ± 1.25^aABα^
		250	23.58 ± 1.47^aAα^	22.78 ± 0.61^abAα^	21.39 ± 1.12^bAα^
Ash	Convection	150	4.67 ± 0.15^aBα^	4.69 ± 0.21^aBα^	4.89 ± 0.18^aBα^
		200	4.65 ± 0.11^bBα^	4.75 ± 0.19^bBα^	5.19 ± 0.13^aAα^
		250	5.16 ± 0.09^aAα^	5.37 ± 0.13^aAα^	5.33 ± 0.19^aAα^
	SHS	150	4.50 ± 0.20^aBα^	4.56 ± 0.16^aBα^	4.77 ± 0.16^aBα^
		200	4.89 ± 0.07^aAα^	4.89 ± 0.16^aAα^	4.74 ± 0.22^aBβ^
		250	5.04 ± 0.05^aAα^	5.06 ± 0.02^aAβ^	5.07 ± 0.04^aAβ^
Carb	Convection	150	36.31 ± 2.38^aAα^	35.73 ± 0.39^aAα^	36.63 ± 4.20^aAα^
		200	36.98 ± 1.04^aAα^	36.57 ± 2.12^aAβ^	35.44 ± 2.07^aAα^
		250	30.66 ± 2.71^aBα^	31.19 ± 2.77^aBα^	29.58 ± 1.88^aBα^
	SHS	150	37.11 ± 0.50^aAα^	39.79 ± 1.37^aAα^	37.25 ± 1.38^aAα^
		200	37.00 ± 0.57^aAα^	36.94 ± 1.06^aBα^	36.50 ± 2.55^aAα^
		250	32.29 ± 1.35^aBα^	30.24 ± 0.11^aCα^	30.11 ± 0.80^aBα^

*Note*: Values are mean ± standard deviation (*n* = 3), expressed in percentage (%). Superscript small letters (^a,b,c^) mean values in a row and superscript capital letters (^A,B,C^) mean values in a column with the same letter are not significantly different (*p* < .05) within the same condition and proximate content values. The same superscript Greek letters (^α,β^) mean values of convection and SHS of the same roasting temperature and time are not significantly different within the same proximate content values.

Abbreviations: Carb, carbohydrate; Temp, temperature.

Unroasted black seeds had a fat content of 32.87 ± 0.55%. Black seed is a well‐known source of therapeutic oil, and although seeds from different origins contain different fat percentages, they typically hold more than 30% fixed oil (Malhotra, 2012; Matthaus & Özcan, 2011). Results indicated an increase in fat content with an increase in roasting time and temperature brought upon by lower moisture content and better extractability (Mazaheri et al., 2019). Better extractability was seemingly facilitated by roasting when plant cell walls were broken down, allowing thorough grinding and better expression of lipids (Holland et al., 2020). Soxhlet extraction method showed no significant difference between SHS and convection in terms of fat content.

Unroasted black seeds had a protein content of 19.74 ± 0.98%. Protein content values in the present study fell within range of values cited in literature as 17.1 (Jan et al., 2019) and 21.66 (Suri et al., 2019). Roasting had little effect on protein content of black seeds (Jan et al., 2019), and neither did the different roasting mediums. Unroasted black seeds had an ash content of 4.21 ± 0.05%, well within the ash content range 2.72%–6.5% from previous studies (Jan et al., 2019; Sharma et al., 2011; Suri et al., 2019). Ash content indicates minerals that are not affected by heating or roasting at these experimental temperatures (Jan et al., 2019; Kour et al., 2021; Silvia et al., 2012). In the present study, a significant increase in ash content was observed in roasted black seeds and when roasting temperatures were increased. This was inerrably brought about by an inverse correlation with moisture content (Tenyang et al., 2022).

Carbohydrate was expressed as total carbohydrate, including fiber (Maclean et al., 2003). Unroasted black seeds had a carbohydrate content of 34.10 ± 1.31%. Carbohydrate values of unroasted black seeds reported in previous studies were mostly similar; 35.39% (Suri et al., 2019) and 35.04% (Mohammed et al., 2016), notwithstanding some outliers, 47.24% (Jan et al., 2019). There was a significant difference in carbohydrate content of black seeds roasted at 250°C for both convection and SHS roasting, however, there were no notable significant differences between SHS and convection roasted black seeds. This significant decrease in carbohydrate content was likely due to the Maillard reaction (Tenyang et al., 2017). Since heat causes the collapse of plant cell walls (Holland et al., 2020), fiber breakdown could have also inferentially resulted in carbohydrate content reduction.

### 3.1 Antioxidant analysis of roasted black seeds

**FIGURE 2:**
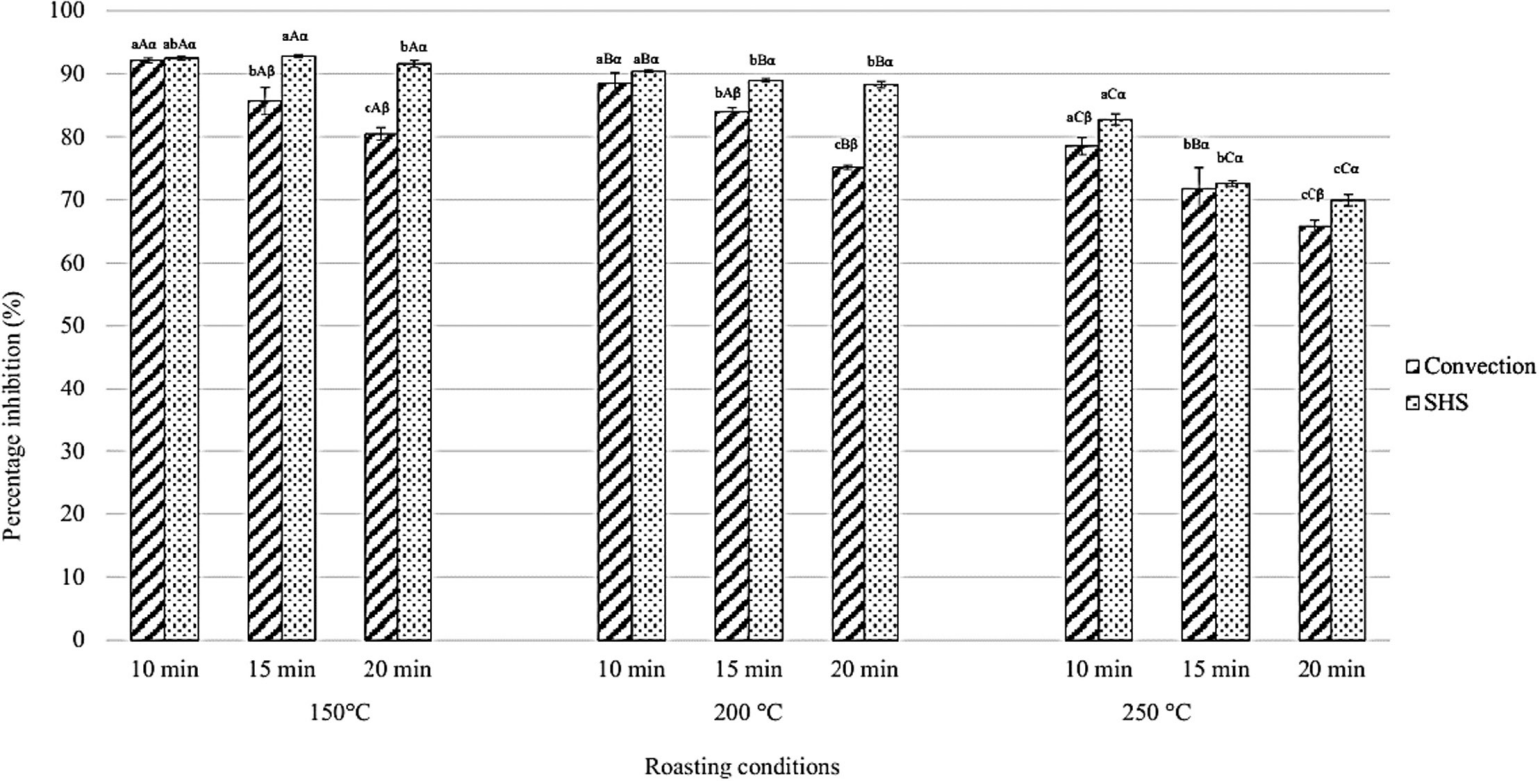
DPPH RSC (in % inhibition) of black seeds roasted via convection and SHS. a,b,c represent significant differences among time within the same temperature and condition; A,B,C represent significant differences among temperature within the same time and condition; α, β represent significant differences between condition of the same roasting temperature and time; all at *p* < .05.

Unroasted black seeds had a radical scavenging value of 90.85 ± 0.97%. DPPH RSC of black seeds significantly decreased as roasting time and temperature were increased in both convection and SHS. However, a more significant decrease in scavenging capacity was observed in the convection roasted sample as opposed to its SHS roasted counterparts (Figure 2). At prolonged roasting times, a significant reduction in DPPH radical scavenging capacity was observed in all three roasting temperatures between convection and SHS roasting, the former being lower than the latter. Contradictory to results from this work, previous reports suggest roasting black seeds increased DPPH RSC (Liang et al., 2018; Varastegani et al., 2018). Liang et al. (2018) roasted black seeds at 180°C and reported a significant increase in DPPH RSC with an increase in roasting time (from 10 to 30 min). However, Liang et al.'s samples were defatted before the antioxidant assay, ergo explaining the incongruence. Similarly, the work of Jan et al. (2019) showed a significant increase in the DPPH RSC of roasted black seeds. The contradiction in Jan et al.'s work was likely caused by a roasting length of no more than 3 min, which would have facilitated the expression of radical‐quenching bioactive compounds but escaped significant thermal damage. At 15 and 20 min of roasting time, SHS roasted black seeds had significantly higher radical scavenging capacity than convection roasted black seeds.

**FIGURE 3:**
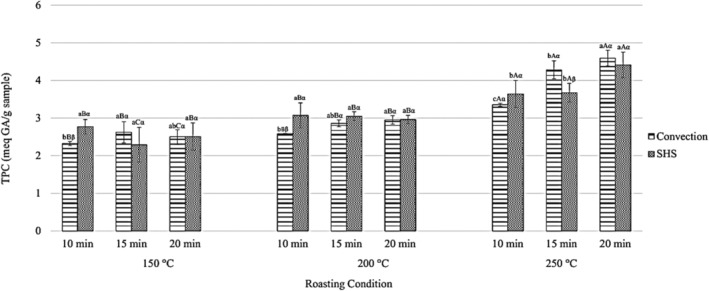
TPC (in mg GAE equivalent/g sample) of black seeds roasted via convection and SHS. a,b,c represent significant differences among time within the same temperature and condition; A,B,C represent significant differences among temperature within the same time and condition; α, β represent significant differences between condition of the same roasting temperature and time; all at *p* < .05.

Unroasted black seeds had a TPC value of 1.71 ± 0.11 mg GAE/g dry weight sample. TPC values of *Nigella sativa* reported in literature were usually higher, 4.1 mg GAE/g (Suri et al., 2019) and 7.4 mg GAE/g (Chauhan et al., 2018). TPC values increased with increased roasting temperatures and roasting time, which was particularly apparent for samples heated at 250°C (Figure 3). Roasting time also contributed to the rise in TPC, which was in agreement with Liang et al. (2018) and Jan et al. (2019). TPC is conversely related to DPPH RSC in this work, a phenomenon that has precedent in Lutterodt et al.'s work (2010), wherein black seed oil samples with the highest TPC value had the lowest DPPH RSC value. Converse relationships between DPPH RSC and TPC values do not indicate a loss of antioxidant potential but rather the formation or increased extractability of phenolic compounds that react differently or do not interact with DPPH radicals (Lutterodt et al., 2010). However, no significant difference was observed in TPC values of black seeds roasted with both SHS and convection.

### 3.2 Oil quality analysis

**FIGURE 4:**
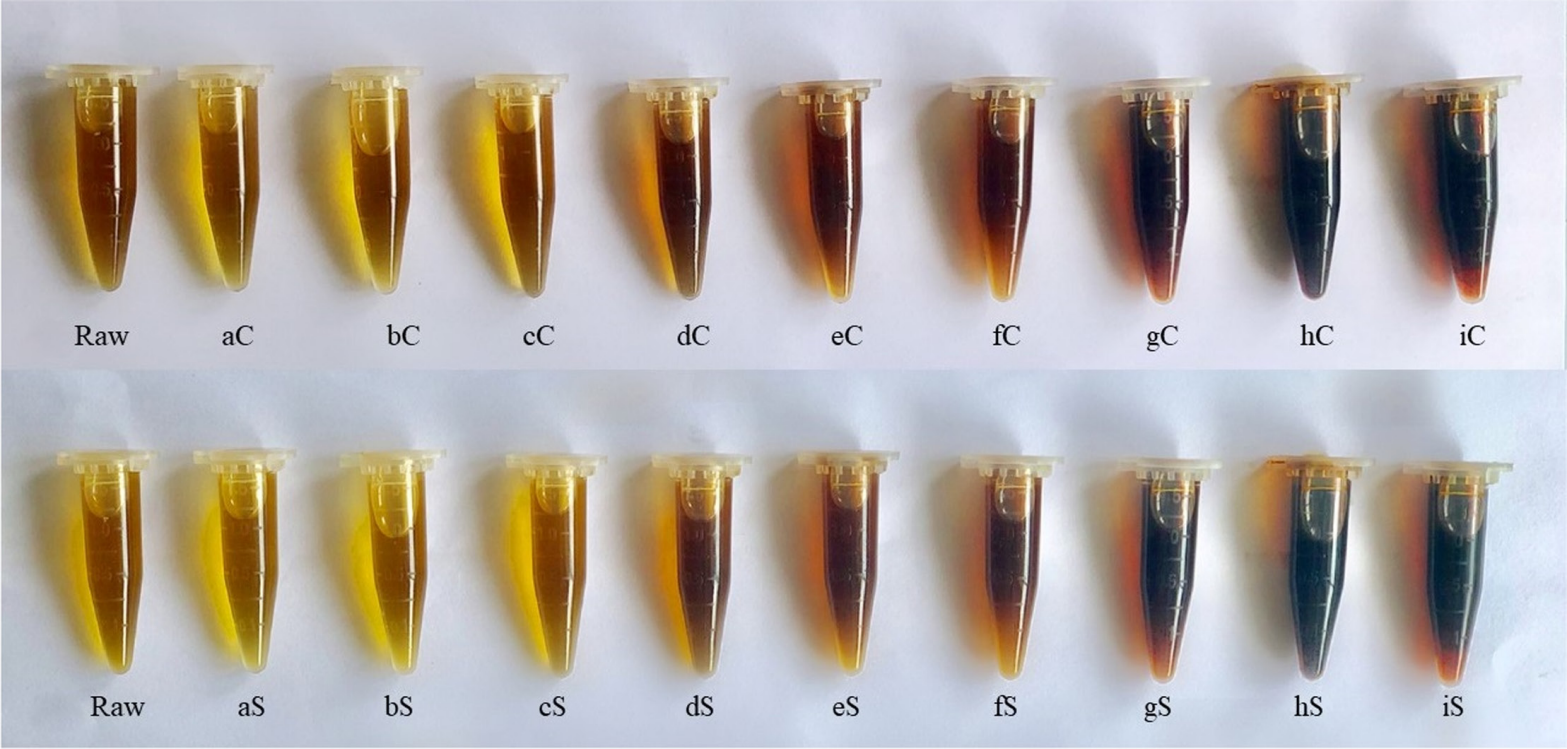
Color difference in oils across different roasting time and temperature. Top row is convection samples designated by aC (150°C, 10 min), bC (150°C, 15 min), cC (150°C, 20 min), dC (200°C, 10 min), eC (200°C, 15 min), fC (200°C, 20 min), gC (250°C, 10 min), hC (250°C, 15 min), and iC (250°C, 20 min). Bottom row is SHS samples designated by aS (150°C, 10 min), bS (150°C, 15 min), cS (150°C, 20 min), dS (200°C, 10 min), eS (200°C, 15 min), fS (200°C, 20 min), gS (250°C, 10 min), hS (250°C, 15 min), and iS (250°C, 20 min).

The importance of measuring oil color lies in observable changes with treated and untreated oil. These changes can indicate impurities in oil that thwart quality and damage marketability (Ramos‐Escudero et al., 2019). Figure 4 shows a clear difference in oil color across roasting times and temperatures (from aC to iC for convection and from aS to iS for SHS). This was further ascertained via a colorimeter. Although atypical, there has been a precedence for using L* a* b* values based on the International Commission on Illumination (CIELAB) to measure edible oil color (Xu, 2003).

According to the International Commission on Illumination for color specification, L* values stand for the lightness of a measured sample (100 lightest, 0 darkest), a* values indicate red‐to‐green color spectrum (positive values indicate redness, negative values indicate greenness), and b* values represent the blue‐to‐yellow spectrum (positive values indicate yellowness, negative values indicate blueness) (Sahin & Sumnu, 2010). L* value of unroasted black seed oil was recorded at 90.30 ± 0.48, indicating a very light‐colored oil, an a* value of −5.96 ± 0.34, indicating a slightly green‐colored oil, and a b* value of 45.72 ± 0.59, indicating a yellow‐colored oil. When the seeds were heated at prolonged and elevated roasting times, the resulting oil was markedly and significantly darker and redder based on its L* a* b* values (Table 2). Darkening of plant oil brought upon by heat is a complex process including, among others, fatty acids, pigments, polymers, and interactions between these compounds that lead to both breakdown and formation of a new composition like aldehydes and ketones (Zzaman et al., 2014). It also indicates impurities capable of causing quality degradation leading to reduced shelf‐life, which is hence considered undesirable (Ramos‐Escudero et al., 2019). This phenomenon has been observed in black seed oils procured via heat‐assisted screw pressing. Black seed oil extracted by screw‐press at 50–100°C was notably dark, with an L* value range of approximately 25–32 (Zzaman et al., 2014). Compared to Table 2, even the lowest L* value was only 49.47 ± 1.04, far lighter than values in Zzaman et al.'s work. At 150°C roasting temperature, L* oil values ranged from 84.75 to 90.83, indicating light color oils. This can be further confirmed in Figure 4. All oils from SHS roasted black seeds (except 200°C, 15 min) had significantly higher L* values than oils from convection roasted black seeds, indicating better color acceptability.

**TABLE 2:** L*, a*, and b* color values of oils from black seeds roasted via convection and SHS.

Color value	Roasting medium	Temp (°C)	Time (min)		
			10	15	20
L*	Convection	150	88.33 ± 1.17^aAβ^	86.25 ± 0.76^bAβ^	84.75 ± 0.28^bAβ^
		200	77.31 ± 1.05^aBβ^	75.87 ± 1.30^aBα^	73.29 ± 1.60^bBβ^
		250	55.56 ± 1.45^aCβ^	53.42 ± 0.22^bCβ^	49.47 ± 1.04^cCβ^
	SHS	150	90.83 ± 0.05^aAα^	90.22 ± 0.07^aAα^	89.77 ± 0.16^aAα^
		200	79.45 ± 0.35^aBα^	76.83 ± 0.83^bBα^	76.56 ± 1.29^bBα^
		250	62.05 ± 0.71^aCα^	55.64 ± 0.94^bCα^	53.09 ± 1.64^cCα^
a*	Convection	150	−4.54 ± 0.97^bCα^	−2.94 ± 0.72^abCα^	−1.80 ± 0.19^aCα^
		200	4.45 ± 0.66^cBα^	6.92 ± 1.68^bBα^	10.40 ± 1.97^aBα^
		250	31.69 ± 1.71^cAα^	35.71 ± 0.76^bAα^	38.72 ± 1.22^aAα^
	SHS	150	−4.75 ± 0.62^aCα^	−4.66 ± 0.43^aCα^	−4.44 ± 0.13^aCβ^
		200	3.10 ± 0.31^bBα^	6.31 ± 0.63^aBα^	6.74 ± 1.15^aBβ^
		250	25.06 ± 1.19^cAβ^	32.19 ± 0.82^bAβ^	35.81 ± 2.47^aAβ^
b*	Convection	150	48.76 ± 2.72^bCα^	53.27 ± 0.96^abCα^	55.27 ± 0.56^aCα^
		200	59.29 ± 6.02^cBβ^	66.43 ± 2.64^bBα^	73.48 ± 2.74^aBα^
		250	89.60 ± 1.04^aAα^	86.72 ± 2.76^abAα^	83.35 ± 1.21^bAβ^
	SHS	150	48.47 ± 0.16^aCα^	48.63 ± 0.73^aCβ^	48.96 ± 0.45^aCβ^
		200	63.36 ± 1.66^bBα^	67.18 ± 0.85^aBα^	67.75 ± 2.49^aBβ^
		250	90.45 ± 1.10^aAα^	89.92 ± 0.93^abAα^	88.02 ± 1.77^bAα^

*Note*: Values are mean ± standard deviation (*n* = 3). Superscript small letters (^a, b, c^) mean values in a row and superscript capital letters (^A, B, C^) mean values in a column with the same letter are not significantly different (*p* < .05) within the same condition and oil color values. The same superscript Greek letters (^α, β^) mean values of convection and SHS of the same roasting temperature and time are not significantly different within the same analysis values.

Abbreviation: Temp, temperature.

Increase in oil yield is typically congruous with heat treatment of oilseeds previously demonstrated in peanuts (Idrus et al., 2017), sesame seeds (Tenyang et al., 2017), and even black seeds (Mazaheri et al., 2019). Results from Table 2 confirmed this as most roasted black seeds yielded significantly more oil than unroasted seeds (17.05 ± 0.28%). All SHS roasted seeds, except samples roasted at 200°C for 20 min, yielded significantly more oil than unroasted seeds. Roasting at 250°C for 20 min (highest temperature, longest time) significantly yielded the most oil (approximately 23.23% for convection and 24.83% for SHS). This phenomenon is attributable to the thermal breakdown of plant cell walls, allowing better oil expression (Mazaheri et al., 2019). Interestingly, the Bligh and Dyer method for oil extraction used here revealed that seeds roasted with SHS had significantly higher oil yield than convection roasted seeds, unlike the Soxhlet extraction method used for proximate content.

According to the Codex Alimentarius, affiliated with the World Health Organization (WHO) and the Food and Agriculture Organization (FAO), there are guidelines for edible fats and oils, albeit unspecified (CXS 19‐1981), amended as late as 2019. These guidelines describe edible oil standards, including methods for expulsion and minimum acid and peroxide values. According to this standard, FFA values of virgin edible oils should not exceed 2.01 (% oleic acid), which means all samples in this work exceed the standard (Codex Alimentarius, 2019). It is, however, noteworthy that black seed edible oils commonly available in the market are mechanically pressed oils as opposed to solvent‐extracted oils due to consumer preference and safety (Zzaman et al., 2014). Considering that information, even cold‐pressed black seed oils can have FFA values of up to 6.15 (% oleic acid) (Mohammed et al., 2016). Measuring the presence of FFA is especially important for measuring oil quality since high levels of FFAs can bring about undesirable sensorial properties by accelerated oil quality deterioration (Gharby et al., 2015). FFA forms due to hydrolysis, further catalyzed by naturally occurring lipase in black seeds (Zzaman et al., 2014). Using the Bligh and Dyer method further contributes to this phenomenon due to using distilled water as part of the solvent mixture. However, unroasted black seed oil value from this work was 5.46 ± 0.41, which is significantly higher than any roasted samples; a result of thermal lipase inactivation during roasting, decreasing enzymatic degradation that usually leads to the formation of FFAs (Mazaheri et al., 2019).

Peroxide value is an important analysis for measuring peroxides formed in oils during the early stages of lipid oxidation (Zzaman et al., 2017). According to the Codex Alimentarius, peroxide values of edible oils should be within 15 milliequivalents of active oxygen per kg oil (Codex Alimentarius, 2019), which means only samples roasted at 250°C (except SHS 250°C, 10 min sample) were within acceptable values. Oil from unroasted seeds had a peroxide value of 57.33 ± 3.06 (meq O_2_/kg of oil). Cold‐pressed black seed oils typically have lower peroxide values ranging from 4.1 to 9.7 (Mazaheri et al., 2019; Mohammed et al., 2016; Suri et al., 2022). However, previous studies have also reported black seed oil peroxide values (meq O_2_/kg of oil) from cold pressed oil at 65.4, with heatless mechanical pressed oil at approximately 25, and Soxhlet extracted oil at 70.8 and 20.7; all of which happened to fall within the range of peroxide value recorded in Table 3 despite being vastly varied (Oubannin et al., 2022; Rokosik et al., 2020). This meant that the elevated ranges of black seed oil peroxide values from Table 3 at 150 and 200°C were due to seed quality rather than extraction method. However, roasting media seemed to have considerably impacted peroxide values. Unlike oils from convection roasted seeds, all oils from SHS roasted black seeds had significantly lower peroxide values than oil from unroasted seeds. Moreover, oils from SHS roasted samples had significantly lower peroxide values than their convection roasted counterparts when roasted at 150 and 200°C. Peroxide values also significantly declined as roasting temperature and time were increased. This is usually regarded as an indication of better oil quality, but, in this case, initially formed peroxides may have been converted into secondary oxidation products. Evaluation via p‐anisidine test thus becomes necessary to confirm this deduction.

**TABLE 3:** Oil quality analysis of black seeds roasted via convection and SHS.

Oil quality analysis	Roasting medium	Temp (°C)	Time (min)		
			10	15	20
Yield (%)	Convection	150	17.16 ± 0.42^bCβ^	17.92 ± 0.27^bCβ^	19.19 ± 0.31^aCβ^
		200	20.23 ± 0.39^bBβ^	20.58 ± 0.42^bBβ^	22.13 ± 0.38^aBα^
		250	22.69 ± 0.79^bAβ^	23.32 ± 0.69^abAβ^	23.23 ± 0.72^aAβ^
	SHS	150	20.17 ± 0.65^bCα^	20.69 ± 0.90^abCα^	21.37 ± 0.47^aCα^
		200	21.71 ± 0.41^bBα^	22.05 ± 0.76^abBα^	22.92 ± 0.69^aBα^
		250	23.61 ± 0.41^bAα^	24.67 ± 0.32^aAα^	24.83 ± 0.57^aAα^
FFA (% oleic acid)	Convection	150	3.15 ± 0.06^aAα^	3.29 ± 0.06^aAα^	3.32 ± 0.10^aAα^
		200	3.32 ± 0.10^aAα^	2.78 ± 0.12^bBβ^	2.68 ± 0.15^bBα^
		250	2.64 ± 0.12^aBα^	2.48 ± 0.12^aCα^	2.28 ± 0.06^bCβ^
	SHS	150	2.91 ± 0.11^aAβ^	3.02 ± 0.11^aAβ^	3.05 ± 0.06^aAβ^
		200	3.25 ± 0.06^aBα^	3.09 ± 0.06^bAα^	2.68 ± 0.06^cBα^
		250	2.61 ± 0.10^aCα^	2.51 ± 0.10^aBα^	2.51 ± 0.10^aCα^
Peroxide (meq O_2_/kg of oil)	Convection	150	84.00 ± 2.00^aAα^	59.33 ± 1.15^bAα^	56.00 ± 2.00^cAβ^
		200	48.67 ± 1.15^aBα^	43.33 ± 1.15^bBα^	36.67 ± 1.15^cBβ^
		250	13.33 ± 1.15^aCβ^	8.67 ± 1.15^bCβ^	8.00 ± 2.00^bCα^
	SHS	150	48.00 ± 2.00^aAβ^	45.33 ± 2.31^aAβ^	40.67 ± 1.15^bAα^
		200	35.33 ± 1.15^aBβ^	28.00 ± 2.00^bBβ^	26.00 ± 2.00^bBα^
		250	16.67 ± 1.15^aCα^	12.67 ± 1.15^bCα^	10.67 ± 1.15^bCβ^
p‐Anisidine	Convection	150	16.25 ± 1.16^bBα^	20.76 ± 2.60^aBα^	22.74 ± 0.83^aBα^
		200	22.30 ± 0.78^aAα^	22.62 ± 2.57^aBα^	23.04 ± 1.73^aBα^
		250	23.00 ± 1.60^bAα^	26.79 ± 1.27^aAα^	28.36 ± 1.12^aAα^
	SHS	150	13.14 ± 1.26^bBβ^	16.46 ± 0.90^aBβ^	18.06 ± 2.02^aBβ^
		200	17.97 ± 1.47^aAβ^	18.53 ± 1.65^aBβ^	20.62 ± 1.39^aBα^
		250	20.45 ± 2.10^bAα^	22.48 ± 1.05^abAβ^	23.73 ± 2.76^aAβ^

*Note*: Values are mean ± standard deviation (*n* = 3). Superscript small letters (^a,b,c^) mean values in a row and superscript capital letters (^A,B,C^) mean values in a column with the same letter are not significantly different (*p* < .05) within the same condition and analysis values. The same superscript Greek letters (^α,β^) mean values of convection and SHS of the same roasting temperature and time are not significantly different.

Abbreviation: Temp, temperature.

Black seed oil from unroasted seeds had a p‐anisidine value of 19.31 ± 1.05 which is higher than typically reported in previous studies, confirming the previous inference that seed quality contributes to high peroxide values. Black seed oil p‐anisidine values reported in literature commonly ranged from 1.42 to 8.52 (Makouie et al., 2021; Ornella et al., 2022; Oubannin et al., 2022; Suri et al., 2019; Turan et al., 2020). Elevated p‐anisidine values confirmed prolonged roasting at high temperatures to be detrimental to black seed oil quality, even considering low peroxide values at higher roasting temperatures and longer roasting time (Table 3). This echoed Suri et al. (2022) work, wherein oil derived from microwave roasted black seeds at increasing power levels resulted in peroxide and p‐anisidine values being inversely correlated, the former on a downward trend while the latter increased. Almost all SHS roasted black seed oils had significantly lower p‐anisidine values than convection roasted black seed oils.

### 3.3 Qualitative analysis of essential oils

A total of 68 compounds were identified—30 in unroasted and the remaining 38—and barring variations were found in roasted black seeds (Table 4). Although unroasted and roasted black seed essential oil profiles were markedly different, major compounds identified in roasted and unroasted black seed essential oils were predominantly aromatics (p‐cymene) and terpenes (4‐terpineol, carvacrol, and longifolene). Thymoquinone was the only exception, wherein it was a major compound in unroasted seeds but not in roasted seeds. These compounds were also found in several previous works on black seed essential oils (Botnick et al., 2012; Edris, 2011). There was also previous report of thymoquinone not detected in any samples (Al Juhaimi et al., 2013). In addition to these major compounds, pyrroles, furans, and pyrazines were also present in roasted black seed essential oils. The past two works on roasted black seed volatile compounds of Kiralan (2012) and Farag et al. (2017) had very differing roasting methods leading to vastly different outcomes. Kiralan's work (2012), comparing microwave roasting (2.450 MHz frequency and 0.45 kW) power to convection oven roasting, found pyrazines, furans, and pyrrole in its volatile compounds, which was akin to the present study. These compounds are commonly formed due to the Maillard reaction, a non‐enzymatic browning process that occurs when foods containing amino acids and sugars are roasted (Kiralan, 2012; Markey, 2019). Through Botnick et al.'s work (2012), it has been proven that monosaccharide sugars glucose, fructose, and galactose were readily present in black seeds. As a result, the occurrence of Maillard reaction when black seeds were roasted was legible. The subsequent presence of Maillard reaction products gives food a characteristic toasted flavor, often pleasant and coveted (Farag et al., 2017; Kiralan, 2012). In the present work, all furans and pyrroles were only detected in samples roasted at 250°C. However, pyrazines (trimethyl pyrazine and 3‐ethyl‐2,5‐dimethyl‐pyrazine) were also detected in other samples. The former was found in both 200 and 250°C roasted samples, while the latter was also detected in 150°C roasted samples. This was the case for both SHS and convection roasted samples. Likewise, despite no different roasting temperatures being used in Kiralan's work (2012), increased microwave roasting times (2, 4, and 8 min) led to the formation of furans, pyrrole, and most pyrazines only after 4 min of roasting time. Meanwhile, in Farag et al.'s work (2017), wherein black seeds were roasted in presumably a regular oven at 120°C for 2 h, no pyrazines or pyrroles were reported. However, unlike the present work, an unidentified furan was reported. The low oven temperature could only be attributed to a difference in either length of roasting time or, less likely, seed variety.

**TABLE 4:** Essential oil profiles of black seeds roasted via convection and SHS.

No	Compound	Condition (roasting media, temperature in °C, and time in minutes)																		
		Unroasted	SHS, 150, 10	SHS, 150, 15	SHS, 150, 20	SHS, 200, 10	SHS, 200, 15	SHS, 200, 20	SHS, 250, 10	SHS, 250, 15	SHS, 250, 20	Conv, 150, 10	Conv, 150, 15	Conv, 150, 20	Conv, 200, 10	Conv, 200, 15	Conv, 200, 20	Conv, 250, 10	Conv, 250, 15	Conv, 250, 20
1.	furfuryl alcohol	n.d.	n.d.	n.d.	n.d.	n.d.	n.d.	n.d.	0.28	0.63	0.37	n.d.	n.d.	n.d.	n.d.	n.d.	n.d.	n.d.	n.d.	n.d.
2.	α‐thujene	1.36	0.76	0.62	0.63	0.42	0.55	4.05	n.d.	n.d.	n.d.	n.d.	n.d.	n.d.	n.d.	n.d.	n.d.	n.d.	n.d.	n.d.
3.	pyrazine, 2,5‐dimethyl‐	n.d.	n.d.	n.d.	n.d.	n.d.	n.d.	n.d.	0.43	1.08	1.40	n.d.	n.d.	n.d.	n.d.	n.d.	n.d.	0.86	1.51	0.44
4.	α‐pinene	0.28	n.d.	n.d.	n.d.	n.d.	n.d.	0.78	n.d.	n.d.	n.d.	n.d.	n.d.	n.d.	n.d.	n.d.	n.d.	n.d.	n.d.	n.d.
5.	sabinene	0.35	0.28	n.d.	n.d.	n.d.	n.d.	0.95	n.d.	n.d.	n.d.	n.d.	n.d.	n.d.	n.d.	n.d.	n.d.	n.d.	n.d.	n.d.
6.	5‐methyl furfural	n.d.	n.d.	n.d.	n.d.	n.d.	n.d.	n.d.	0.39	0.86	0.40	n.d.	n.d.	n.d.	n.d.	n.d.	n.d.	n.d.	n.d.	n.d.
7.	β‐pinene	0.99	0.69	0.51	0.47	0.25	0.31	1.90	n.d.	n.d.	n.d.	n.d.	n.d.	n.d.	n.d.	n.d.	n.d.	n.d.	n.d.	n.d.
8.	phenyl alcohol	n.d.	n.d.	n.d.	n.d.	n.d.	n.d.	n.d.	0.33	0.53	1.43	n.d.	n.d.	n.d.	n.d.	n.d.	n.d.	n.d.	2.41	1.21
9.	pyrazine, 2‐ethyl‐, 6‐methyl‐	n.d.	n.d.	n.d.	n.d.	n.d.	n.d.	n.d.	0.27	0.59	0.72	n.d.	n.d.	n.d.	n.d.	n.d.	n.d.	0.49	1.09	n.d.
10.	trimethylpyrazine	n.d.	n.d.	n.d.	n.d.	0.30	n.d.	n.d.	1.01	1.71	1.82	n.d.	n.d.	n.d.	n.d.	0.29	0.38	0.83	2.83	0.61
11.	α‐terpinene	0.28	0.49	0.35	0.32	0.26	n.d.	0.33	n.d.	n.d.	n.d.	n.d.	n.d.	n.d.	n.d.	n.d.	n.d.	n.d.	n.d.	n.d.
12.	p‐cymene	45.66	36.02	27.23	27.65	23.74	18.97	60.16	18.60	39.10	27.01	13.08	15.99	1.02	n.d.	0.96	1.21	22.00	18.56	14.27
13.	limonene	n.d.	1.89	1.48	1.46	1.12	0.83	2.77	0.61	1.37	0.80	0.56	0.72	n.d.	n.d.	n.d.	n.d.	0.65	n.d.	0.41
14.	eucalyptol	n.d.	0.19	n.d.	0.20	n.d.	n.d.	0.17	n.d.	n.d.	n.d.	n.d.	n.d.	n.d.	n.d.	n.d.	n.d.	n.d.	n.d.	n.d.
15.	γ‐terpinene	0.56	4.37	3.57	3.63	3.64	1.63	1.47	0.54	1.08	2.65	1.31	1.72	n.d.	n.d.	n.d.	n.d.	n.d.	n.d.	1.65
16.	trans‐sabinene hydrate	0.40	0.46	0.49	0.52	0.43	0.34	0.18	0.48	0.15	n.d.	0.52	0.54	n.d.	0.32	0.46	0.54	n.d.	n.d.	n.d.
17.	pyrazine, 3‐ethyl‐2,5‐dimethyl‐	n.d.	n.d.	n.d.	0.37	0.39	0.32	n.d.	0.92	0.97	1.34	n.d.	0.26	n.d.	0.36	0.66	0.86	0.78	2.68	1.13
18.	terpinolene	n.d.	0.18	0.29	n.d.	n.d.	n.d.	n.d.	n.d.	n.d.	n.d.	n.d.	n.d.	n.d.	n.d.	n.d.	n.d.	n.d.	n.d.	n.d.
19.	pyrrole	n.d.	n.d.	n.d.	n.d.	n.d.	n.d.	n.d.	n.d.	0.20	1.02	n.d.	n.d.	n.d.	n.d.	n.d.	n.d.	1.08	2.86	1.65
20.	linalool	1.97	2.26	1.99	2.15	2.34	2.32	2.23	n.d.	n.d.	n.d.	1.67	1.80	n.d.	n.d.	0.68	0.79	n.d.	n.d.	n.d.
21.	p‐cymenene	n.d.	n.d.	n.d.	n.d.	n.d.	n.d.	n.d.	2.32	4.34	4.61	n.d.	n.d.	n.d.	n.d.	n.d.	n.d.	3.79	1.32	2.29
22.	guaiacol	n.d.	n.d.	n.d.	n.d.	n.d.	n.d.	n.d.	n.d.	n.d.	n.d.	n.d.	n.d.	n.d.	n.d.	n.d.	n.d.	0.49	2.94	0.90
23.	cis beta terpineol	0.37	0.54	0.61	0.63	0.56	0.45	0.19	0.59	0.22	n.d.	0.71	0.71	0.54	0.59	0.71	0.80	n.d.	n.d.	n.d.
24.	cyclopentapyrazine <5‐methyl‐, 6,7‐dihydro‐, 5(h)‐>	n.d.	n.d.	n.d.	n.d.	n.d.	n.d.	n.d.	n.d.	n.d.	0.75	n.d.	n.d.	n.d.	n.d.	n.d.	n.d.	n.d.	1.78	0.59
25.	2,6‐dimethyl‐3,5,7‐octatriene‐2‐ol	0.80	0.80	0.72	0.81	0.95	1.56	0.92	0.68	0.63	n.d.	0.92	0.90	0.23	0.58	1.01	1.18	3.15	n.d.	0.35
26.	trans‐3(10)‐caren‐2‐ol	n.d.	n.d.	n.d.	n.d.	n.d.	n.d.	n.d.	n.d.	n.d.	n.d.	n.d.	n.d.	n.d.	0.34	0.40	0.35	n.d.	n.d.	n.d.
27.	4‐terpineol	4.60	6.35	7.82	8.15	6.85	8.33	3.46	10.31	6.95	4.26	9.33	9.16	6.79	7.79	10.17	13.14	5.51	3.63	4.33
28.	furfuryl pyrrole	n.d.	n.d.	n.d.	n.d.	n.d.	n.d.	n.d.	n.d.	n.d.	0.51	n.d.	n.d.	n.d.	n.d.	n.d.	n.d.	n.d.	1.05	0.32
29.	p‐cymene‐8‐ol	1.17	1.45	1.82	1.93	1.92	1.98	0.88	3.35	2.40	1.75	2.21	2.22	2.55	2.69	3.28	4.28	1.63	1.44	1.76
30.	cis‐limonene oxide	1.39	1.44	1.46	1.60	1.67	3.63	1.82	0.85	0.71	n.d.	1.87	1.70	0.79	1.12	1.89	2.43	n.d.	n.d.	n.d.
31.	4‐methyl salicylaldehyde	n.d.	0.27	0.30	0.34	0.21	0.27	n.d.	n.d.	n.d.	0.33	0.34	0.33	n.d.	n.d.	0.23	0.24	n.d.	1.60	0.91
32.	α‐terpinyl methyl ether	n.d.	0.18	0.20	0.21	0.21	0.29	0.15	0.28	0.54	0.62	n.d.	0.21	n.d.	n.d.	n.d.	n.d.	n.d.	0.57	0.31
33.	trans pinocarveol	n.d.	0.74	0.79	0.85	1.06	1.63	0.38	2.50	1.62	1.66	1.13	1.12	1.28	1.73	1.82	1.12	n.d.	0.73	0.60
34.	thymoquinone	9.20	1.41	1.06	1.10	0.67	0.38	0.19	0.42	0.32	0.33	2.67	1.56	2.08	4.50	1.17	0.75	n.d.	n.d.	n.d.
35.	4(1h)‐Quinazolinone	n.d.	n.d.	n.d.	n.d.	n.d.	n.d.	n.d.	0.51	0.68	1.93	n.d.	n.d.	n.d.	n.d.	n.d.	n.d.	0.67	4.65	2.62
36.	1‐(5‐methylfurfuryl)‐pyrrole	n.d.	n.d.	n.d.	n.d.	n.d.	n.d.	n.d.	n.d.	n.d.	0.33	n.d.	n.d.	n.d.	n.d.	n.d.	n.d.	n.d.	1.20	0.67
37.	trans shisool	n.d.	n.d.	n.d.	n.d.	n.d.	n.d.	n.d.	0.51	0.51	1.35	n.d.	n.d.	n.d.	n.d.	n.d.	n.d.	0.49	1.23	0.93
38.	bornyl acetate	0.60	0.68	0.83	0.77	0.98	1.14	0.51	1.23	0.94	1.02	1.11	1.02	1.05	1.23	1.73	2.13	0.81	n.d.	0.64
39.	4‐allyl anisole	n.d.	0.30	0.27	0.28	0.33	0.48	0.17	0.46	0.17	0.34	0.38	0.34	0.25	0.66	0.55	0.57	n.d.	n.d.	n.d.
40.	2‐undecanone	n.d.	n.d.	0.25	0.22	0.24	0.32	n.d.	n.d.	n.d.	n.d.	0.29	0.28	0.29	0.26	0.47	0.44	n.d.	n.d.	n.d.
41.	indene‐1,7(4h)‐dione	0.54	n.d.	n.d.	n.d.	n.d.	n.d.	n.d.	n.d.	n.d.	n.d.	n.d.	n.d.	n.d.	n.d.	n.d.	n.d.	n.d.	n.d.	n.d.
42.	thymol	0.41	0.50	0.72	0.68	0.76	0.17	n.d.	0.87	n.d.	0.57	0.90	0.87	1.58	2.33	1.63	1.94	0.91	1.82	1.42
43.	carvacrol	11.97	15.65	19.53	19.13	18.97	19.36	6.56	18.61	10.73	15.72	26.16	24.87	40.41	32.13	34.24	25.25	20.69	26.13	31.02
44.	α‐longipinene	1.93	2.81	3.07	3.09	4.06	4.34	1.74	4.02	3.44	2.78	3.61	3.70	1.97	3.45	3.70	4.80	3.52	1.29	2.26
45.	2‐(2′‐furyl)‐5‐methylpyrazine	n.d.	n.d.	n.d.	n.d.	n.d.	n.d.	n.d.	n.d.	n.d.	0.40	n.d.	n.d.	n.d.	n.d.	n.d.	n.d.	n.d.	0.80	0.55
46.	2‐methylchromone	n.d.	n.d.	n.d.	n.d.	n.d.	n.d.	n.d.	n.d.	n.d.	0.36	n.d.	n.d.	n.d.	n.d.	n.d.	n.d.	n.d.	1.16	0.91
47.	longicyclene	n.d.	0.19	0.22	0.23	0.29	n.d.	n.d.	n.d.	n.d.	n.d.	0.26	0.26	0.23	0.26	0.32	0.35	n.d.	n.d.	n.d.
48.	limonene oxide	n.d.	0.96	1.15	1.19	0.98	n.d.	n.d.	n.d.	n.d.	n.d.	1.38	1.56	3.42	2.97	1.64	2.07	n.d.	n.d.	0.47
49.	2‐allyl‐4‐methylphenol	n.d.	n.d.	n.d.	n.d.	n.d.	n.d.	n.d.	n.d.	n.d.	n.d.	n.d.	n.d.	n.d.	n.d.	n.d.	n.d.	0.74	n.d.	0.40
50.	α‐amorphene	0.27	0.31	0.37	0.38	0.46	0.49	0.14	0.45	0.32	0.47	0.46	0.44	0.40	0.49	0.49	0.54	n.d.	n.d.	0.34
51.	longifolene	8.99	11.57	12.94	12.71	17.21	19.29	6.86	17.80	13.76	14.04	15.96	15.92	13.61	17.89	17.99	17.70	16.17	8.49	11.81
52.	trans beta caryophyllene	n.d.	0.20	0.25	0.23	0.34	0.51	n.d.	0.32	n.d.	n.d.	0.28	0.30	0.41	0.42	0.33	0.29	n.d.	n.d.	n.d.
53.	butyl hydroxy anisole	n.d.	n.d.	n.d.	n.d.	n.d.	n.d.	n.d.	n.d.	n.d.	n.d.	n.d.	n.d.	n.d.	n.d.	n.d.	n.d.	n.d.	0.46	0.50
54.	2‐tridecanone	0.37	0.39	0.54	0.60	0.74	0.94	0.22	0.83	0.47	0.86	0.69	0.83	1.65	1.47	1.16	1.34	0.52	0.60	0.79
55.	beta bisabolene	n.d.	0.16	n.d.	n.d.	0.30	0.50	n.d.	0.34	n.d.	n.d.	0.27	0.27	0.50	0.53	0.41	0.34	n.d.	n.d.	n.d.
56.	thymohydroquinone	0.83	0.25	n.d.	n.d.	n.d.	n.d.	n.d.	n.d.	n.d.	n.d.	n.d.	n.d.	n.d.	n.d.	n.d.	n.d.	n.d.	n.d.	n.d.
57.	tridec‐(2e)‐enal	n.d.	0.25	0.37	0.41	0.44	0.64	n.d.	0.45	n.d.	n.d.	0.44	0.52	0.96	0.73	0.54	0.69	n.d.	n.d.	n.d.
58.	trans‐2‐dodecen‐1‐ol	n.d.	n.d.	0.21	0.20	n.d.	0.29	n.d.	n.d.	n.d.	n.d.	0.34	0.30	0.64	0.35	n.d.	n.d.	n.d.	n.d.	n.d.
59.	humulane‐1,6‐dien‐3‐ol	0.37	0.28	0.36	0.41	0.48	0.56	n.d.	0.56	0.31	0.50	0.50	0.55	1.03	0.91	0.67	0.83	0.97	0.46	0.60
60.	ethanone, 1‐cyclododecyl‐	0.19	0.15	0.21	0.22	0.24	0.28	n.d.	0.30	n.d.	n.d.	0.31	0.29	0.65	0.57	0.38	0.43	n.d.	n.d.	0.31
61.	2,3‐dihydrofarnesol	n.d.	n.d.	n.d.	n.d.	n.d.	n.d.	n.d.	n.d.	n.d.	n.d.	n.d.	0.18	0.49	0.51	0.27	0.27	n.d.	n.d.	n.d.
62.	citronellal	n.d.	0.40	0.71	0.62	0.60	0.75	n.d.	0.50	n.d.	n.d.	0.81	0.82	1.64	1.20	0.80	0.93	0.81	n.d.	n.d.
63.	5‐dodecen‐1‐ol, acetate	n.d.	n.d.	0.19	n.d.	n.d.	0.23	n.d.	0.26	n.d.	n.d.	0.28	0.25	0.48	0.42	0.28	0.24	0.47	n.d.	0.34
64.	sandaracopimaradiene	n.d.	1.15	1.05	1.00	1.04	1.47	0.23	1.34	0.68	1.25	1.22	1.33	2.55	1.73	1.43	1.72	2.49	2.05	2.59
65.	tetradec‐(9e)‐en‐1‐yl acetate	0.47	0.29	0.43	0.40	0.42	0.96	n.d.	1.43	0.60	1.75	0.80	0.53	1.14	1.00	0.63	0.66	1.13	0.41	0.89
66.	citronellyl butyrate	1.11	1.03	2.40	1.91	1.74	1.27	0.21	1.57	0.63	1.06	2.87	2.45	3.08	3.06	2.91	3.62	3.06	n.d.	2.85
67.	butyric acid tetradecyl ester	0.31	0.17	0.35	0.23	0.25	0.32	n.d.	0.41	n.d.	0.34	0.53	0.41	0.81	0.65	0.44	0.56	0.89	0.51	0.77
68.	citronellylacetone	2.29	1.52	2.28	2.07	2.16	1.90	0.39	2.07	0.76	1.15	3.82	2.78	5.48	4.76	3.25	4.22	4.40	1.74	2.59

*Note*: Values are presented as percentage area (%) in order of retention time.

Abbreviations: Conv, convection; n.d., not detected.

From Table 4, lesser compounds, specifically indene‐1,7(4h)‐dione and thymohydroquinone, were completely undetected in all roasted samples. The latter was perceptible in only a single SHS roasted sample (150°C, 10 min). Additionally, α‐thujene, β‐pinene, α‐terpinene, linalool, 2‐undecanone, and longicyclene, all compounds present in unroasted black seed, were made untraceable after being roasted at 250°C, regardless of roasting media. Comparably, in Kiralan's work (2012), some compounds like α‐pinene and α‐terpinene were untraceable in 4 and 8 min microwave roasted samples and convection roasted samples, while carvacrol was completely void in all roasted samples. Otherwise, in the present work (Table 4), there were also many minor functional compounds only seen in roasted samples, for instance, limonene, 4‐methyl salicylaldehyde, α‐terpinyl methyl ether, 4‐allyl anisole, and longicyclene, to name a few. This makes an encouraging argument to support roasting as a necessary processing step for black seeds since roasting leads to the expression and bioavailability of volatile compounds (Sruthi et al., 2021).

Thymoquinone is a major phytochemical often associated with black seed consumption benefits, a key component of black seed essential oil (Rokosik et al., 2020). Farag et al. (2017) hypothesized that thymoquinone detected in samples could be lost during oil extraction, wherein mechanical pressing is commonplace. They further confirmed this hypothesis by comparing volatiles from commercial nigella seed oil and from seeds roasted for 2 h at 120°C. Both samples recorded significantly lower thymoquinone content than unroasted seeds. Black seed oil had far less thymoquinone than even roasted seeds. Other prior work revealed that thymoquinone is also adversely affected when seeds are heated to more than 200°C (Agbaria et al., 2015). Microwave roasting also caused a significant decrease in thymoquinone content despite a brief 2‐min heating time (Kiralan, 2012). From Table 4, surprisingly, thymoquinone was present in all SHS roasted samples. However, thymoquinone was undetected in convection roasted samples in samples roasted at 250°C for 20 min. Convection roasting also led to several minor compounds being rendered untraceable, namely, α‐thujene, β‐pinene, and α‐terpinene, which were otherwise still present in several SHS roasted samples and the unroasted sample as well. This observation may support previous claims that lack of oxygen in SHS contributed to the preservation of bioactive compounds in roasted products (Shan et al., 2015).

## 4 CONCLUSION

Roasting proved crucial to enhance certain qualities of black seeds, for instance, moisture loss, improved antioxidant capacity, and formation of certain volatile compounds that enhance flavor. However, despite substantial moisture loss and increase in TPC, roasting black seeds at 250°C caused substantial loss of RSC, enhanced lipid oxidation, contributed to adverse changes in essential oil, namely loss of thymoquinone, and ultimately did more harm than good. Longer roasting time also contributed to this phenomenon. Regarding roasting media, SHS was significantly better than convection roasting in multiple instances, namely DPPH RSC, oil color, oil yield, peroxide value, p‐anisidine value, and preservation of thymoquinone. In all other physicochemical analyses performed, no significant difference was detected between both roasting media meant that convection roasting did not outperform SHS in any other quality assessments.

## AUTHOR CONTRIBUTIONS

**K.V. Harivaindaran:** Data curation (equal); investigation (equal); resources (equal); software (equal); visualization (equal); writing – original draft (equal). **Nguyễn Hữu Tiến:** Investigation (equal); methodology (equal); resources (equal); visualization (equal); writing – original draft (equal). **Toàn Nguyễn Song Đinh:** Data curation (equal); investigation (equal); validation (equal); visualization (equal); writing – original draft (equal). **Hayati Samsudin:** Conceptualization (equal); project administration (equal); supervision (equal). **Fazilah Ariffin:** Conceptualization (equal); data curation (equal); funding acquisition (equal); methodology (equal); project administration (equal); supervision (equal); validation (equal). **Abdorreza Mohammadi Nafchi:** Data curation (equal); formal analysis (equal); methodology (equal); project administration (equal); validation (equal); visualization (equal); writing – review and editing (equal).

## FUNDING INFORMATION

This research was funded by RUI grant (1001.PTEKIND.8011042). The authors acknowledge School of Industrial Technology, Universiti Sains Malaysia, for staff, lab assistance, and facilities provided.

## CONFLICT OF INTEREST STATEMENT

The authors declare no conflict of interest.

## ETHICS STATEMENT

This study does not involve any human or animal testing.

## Data Availability

The data that support the findings of this study are available from the corresponding author upon reasonable request.
